# Supplementary material for: Thermal phenotypic plasticity of pre- and post-copulatory male harm buffers sexual conflict in wild Drosophila melanogaster
Source: eLife. 2023 Apr 27;12:e84759. doi: 10.7554/eLife.84759 (PMC10191624; doi:10.7554/eLife.84759)
Supplement: Table 1—source data 1. — (a) Polyandry – Monogamy contrast table for each temperature level for female fitness components. (b) Polyandry – Monogamy contrast table for each temperature level for underlying behavioral mechanisms. Test from generalized linear models (GLMs) fitted with temperature as factor. Note that using Tukey’s post hoc yielded qualitatively identical results from running models separately for each temperature. [file elife-84759-table1-data1.docx]

**Table 1 – source data 1**.

a)

| ***T*°C** | ***LRS*** | | | | ***Reproductive ageing*** | | | | ***Actuarial ageing*** | | | |
| --- | --- | --- | --- | --- | --- | --- | --- | --- | --- | --- | --- | --- |
|  | *T ratio* | *Df* | *p* | *Estimate +/- SE* | *T ratio* | *Df* | *p* | *Estimate +/- SE* | *T ratio* | *Df* | *p* | *Estimate +/- SE* |
| 20° | 2.31 | 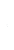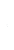422 | **0.022** | 3.73±1.6 | -2.9 | 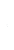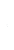422 | **0.004** | -16±5.5 | 8.05 | 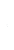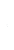425 | **<0.001** | 14.9±1.8 |
| 24° | 3.66 | 422 | **<0.001** | 5.99±1.6 | -6.19 | 422 | **<0.001** | -34.4±5.6 | 5.2 | 425 | **<0.001** | 9.69±1.8 |
| 28° | 1.35 | 422 | 0.177 | 2.27±1.7 | -4.16 | 422 | **<0.001** | -23.8±5.7 | 3.12 | 425 | **0.002** | 5.95±1.9 |

b)

| ***T*°C** | ***Courtship rate*** | | | | ***Rejection rate*** | | | |
| --- | --- | --- | --- | --- | --- | --- | --- | --- |
|  | *T ratio* | *Df* | *p* | *Estimate +/- SE* | *T ratio* | *Df* | *p* | *Estimate +/- SE* |
| 20° | 0.14 | 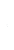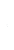438 | 0.891 | 0.25±1.8 | -0.48 | 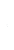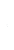438 | 0.628 | -0.07±0.14 |
| 24° | -5.25 | 438 | **<0.001** | -9.66±1.8 | -3.6 | 438 | **<0.001** | -0.5±0.14 |
| 28° | -5.91 | 438 | **<0.001** | -11.0±1.9 | -4.15 | 438 | **<0.001** | -0.62±0.15 |
