# Supplementary material for: Thermal phenotypic plasticity of pre- and post-copulatory male harm buffers sexual conflict in wild Drosophila melanogaster
Source: eLife. 2023 Apr 27;12:e84759. doi: 10.7554/eLife.84759 (PMC10191624; doi:10.7554/eLife.84759)
Supplement: Table 1—source data 2. — (a) Summary statistics from Cox PH survival full model. (b) Summary statistics from fitting separate Cox PH models for each temperature level due to a significant interaction between temperature and mating system. (c) Polyandry – Monogamy contrast table from Tukey’s post hoc for each temperature level from Cox PH survival model fitted with temperature as factor. p-values from Cox HP models are computed using ANOVA type III, LR test. Note that using Tukey’s post hoc yielded qualitatively identical results from running models separately for each temperature. The corresponding survival plot is plotted in Figure 4—figure supplement 1 . [file elife-84759-table1-data2.docx]

**Table 1 – source data 2.**

a)

| ***Effect*** | ***Chisq*** | ***Df*** | ***p value**** |
| --- | --- | --- | --- |
| *Temperature * Mating System* | 7.18 | 1 | **0,007** |
| *Mating System* | 16.14 | 1 | **<0,001** |
| *Temperature* | 62.60 | 1 | **<0.001** |

* p-values were corrected for multiple testing using BH correction

b)

| ***T°*** | ***Chisq*** | ***Df*** | ***p value**** | ***Estimate (95% CI)*** |
| --- | --- | --- | --- | --- |
| 20°C | 43.42 | 1 | **<0.001** | 1.87 (1.55 - 2.26) |
| 24°C | 40.06 | 1 | **<0.001** | 1.83 (1.52 - 2.22) |
| 28°C | 18.21 | 1 | **<0.001** | 1.46 (1.23 - 1.74) |

* p-values were corrected for multiple testing using BH correction

c)

| ***T°*** | ***Estimate*** | ***SE*** | ***Df*** | ***T ratio*** | ***p value*** |
| --- | --- | --- | --- | --- | --- |
| 20°C | 1.27 | 0.18 | Inf | 6.98 | **<0.001** |
| 24°C | 1.29 | 0.17 | Inf | 7.36 | **<0.001** |
| 28°C | 0.59 | 0.17 | Inf | 3.47 | **<0.001** |
