# Supplementary material for: Thermal phenotypic plasticity of pre- and post-copulatory male harm buffers sexual conflict in wild Drosophila melanogaster
Source: eLife. 2023 Apr 27;12:e84759. doi: 10.7554/eLife.84759 (PMC10191624; doi:10.7554/eLife.84759)
Supplement: Table 3—source data 1. — (a) High – low sperm competition risk contrast table for each temperature level. (b) Long – short treatment duration contrast table for each temperature level. (c) High – low sperm competition risk contrast table for each treatment duration. Test from generalized linear models (GLMs) fitted with temperature as factor. Note that using Tukey’s post hoc yielded qualitatively identical results from running models separately for each temperature or treatment duration. [file elife-84759-table3-data1.docx]

**Table 3 – source data 1**.

a)

| ***T*°C** | ***Mating duration**** | | | | ***Remating latency**** | | | |
| --- | --- | --- | --- | --- | --- | --- | --- | --- |
|  | *T ratio* | *Df* | *p* | *Estimate +/- SE* | *T ratio* | *Df* | *p* | *Estimate +/- SE* |
| 20° | 1.7 | 1232 | 0.08 | 0.05±0.02 | 1.109 | 1087 | 0.268 | 66.7±60.1 |
| 24° | 2.98 | 1232 | **0.002** | 0.08±0.02 | -0.071 | 1087 | 0.943 | -4.5±62.6 |
| 28° | 5.74 | 1232 | **<0.001** | 0.17±0.02 | 2.969 | 1087 | **0.003** | 183.2±61.7 |

*Results are averaged over the levels of treatment duration

b)

| ***T*°C** | ***Mating duration**** | | | | ***Remating latency**** | | | |
| --- | --- | --- | --- | --- | --- | --- | --- | --- |
|  | *T ratio* | *Df* | *p* | *Estimate +/- SE* | *T ratio* | *Df* | *p* | *Estimate +/- SE* |
| 20° | -1.4 | 1232 | 0.143 | -0.04±0.03 | -0.022 | 1087 | 0.982 | -1.32±60.1 |
| 24° | -1.9 | 1232 | **0.046** | -0.05±0.03 | -0.216 | 1087 | 0.828 | -13.5±62.6 |
| 28° | -0.8 | 1232 | 0.41 | -0.02±0.03 | -3.259 | 1087 | **0.001** | -201.1±61.7 |

*Results are averaged over the levels of sperm competition risk

c)

| ***Treatment***  ***duration*** | ***Mating duration**** | | | |
| --- | --- | --- | --- | --- |
|  | *T ratio* | *Df* | *p* | *Estimate +/- SE* |
| Short (48 hours) | 2.41 | 1232 | **0.016** | 0.06±0.02 |
| Long (13 days) | 6.33 | 1232 | **<0.001** | 0.143±0.02 |

*Results are averaged over the levels of temperature
