# Supplementary material for: Thermal phenotypic plasticity of pre- and post-copulatory male harm buffers sexual conflict in wild Drosophila melanogaster
Source: eLife. 2023 Apr 27;12:e84759. doi: 10.7554/eLife.84759 (PMC10191624; doi:10.7554/eLife.84759)
Supplement: Table 4—source data 1. — Note that using temperature as a factor yielded qualitatively identical results than treating it as a continuous covariable. p-values from Hurdel model are computed using ANOVA type III, Wald test. Corresponding data is plotted in Figure 6—figure supplement 2. [file elife-84759-table4-data1.docx]

**Table 4 – source data 1.**

| ***Effect*** | ***Chisq*** | ***Df*** | ***p value*** |
| --- | --- | --- | --- |
| *SCR* | 0.0016 | 1 | 0.968 |
| *Temperature* | 2.7419 | 2 | 0.253 |
| *Treatment duration* | 9.0076 | 1 | **0.002** |
| *SCR*Treatment duration* | 0.039 | 1 | 0.843 |
| *SCR*Temperature* | 1.9116 | 2 | 0.384 |
| *Temperature*Treatment duration* | 2.7871 | 2 | 0.248 |
