# Supplementary figures and images for: Thermal phenotypic plasticity of pre- and post-copulatory male harm buffers sexual conflict in wild Drosophila melanogaster
Source: eLife. 2023 Apr 27;12:e84759. doi: 10.7554/eLife.84759 (PMC10191624; doi:10.7554/eLife.84759)

**Table 4 – source data 2**.

| ***T*°C** | ***Total of adults*** | | | |
| --- | --- | --- | --- | --- |
| *T ratio* | *Df* | *p* | *Estimate +/- SE* |
| 20° | 0.74 | 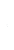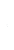947 | 0.453 | 2.81±3.76 |
| 24° | 2.17 | 947 | **0.029** | 8.19±3.76 |
| 28° | 2.18 | 947 | **0.028** | 8.44±3.86 |

Supplement: Table 4—source data 2. — Short (48 hr) – Long (13 days) treatment duration contrast table for each temperature level. Test from generalized linear models (GLMs) fitted with temperature as factor. Note that using Tukey’s post hoc yielded qualitatively identical results from running models separately for each temperature. [file elife-84759-table4-data2.docx]
